# Risk stratification of thymic epithelial tumors based on peritumor CT radiomics and semantic features

**DOI:** 10.1186/s13244-024-01798-2

**Published:** 2024-10-22

**Authors:** Lin Zhang, Zhihan Xu, Yan Feng, Zhijie Pan, Qinyao Li, Ai Wang, Yanfei Hu, Xueqian Xie

**Affiliations:** 1 Radiology Department, Shanghai General Hospital, Shanghai Jiao Tong University School of Medicine, Shanghai, China; 2 Siemens Healthineers Ltd., Shanghai, China; 3 Radiology Department, Shanghai General Hospital, University of Shanghai for Science and Technology, Shanghai, China; 4 Radiology Department, Jiading District Jiangqiao Hospital, Shanghai, China

**Keywords:** Thymic epithelial tumor, Tomography (X-ray computed), Radiomics, Nomograms

## Abstract

**Objectives:**

To develop and validate nomograms combining radiomics and semantic features to identify the invasiveness and histopathological risk stratification of thymic epithelial tumors (TET) using contrast-enhanced CT.

**Methods:**

This retrospective multi-center study included 224 consecutive cases. For each case, 6764 intratumor and peritumor radiomics features and 31 semantic features were collected. Multi-feature selections and decision tree models were performed on radiomics features and semantic features separately to select the most important features for Masaoka–Koga staging and WHO classification. The selected features were then combined to create nomograms for the two systems. The performance of the radiomics model, semantic model, and combined model was evaluated using the area under the receiver operating characteristic curves (AUCs).

**Results:**

One hundred eighty-seven cases (56.5 years ± 12.3, 101 men) were included, with 62 cases as the external test set. For Masaoka–Koga staging, the combined model, which incorporated five peritumor radiomics features and four semantic features, showed an AUC of 0.958 (95% CI: 0.912–1.000) in distinguishing between early-stage (stage I/II) and advanced-stage (III/IV) TET in the external test set. For WHO classification, the combined model incorporating five peritumor radiomics features and two semantic features showed an AUC of 0.857 (0.760–0.955) in differentiating low-risk (type A/AB/B1) and high-risk (B2/B3/C) TET. The combined models showed the most effective predictive performance, while the semantic models exhibited comparable performance to the radiomics models in both systems (*p* > 0.05).

**Conclusion:**

The nomograms combining peritumor radiomics features and semantic features could help in increasing the accuracy of grading invasiveness and risk stratification of TET.

**Critical relevance statement:**

Peripheral invasion and histopathological type are major determinants of treatment and prognosis of TET. The integration of peritumoral radiomics features and semantic features into nomograms may enhance the accuracy of grading invasiveness and risk stratification of TET.

**Key Points:**

- Peritumor region of TET may suggest histopathological and invasive risk.
- Peritumor radiomic and semantic features allow classification by Masaoka–Koga staging (AUC: 0.958).
- Peritumor radiomic and semantic features enable the classification of histopathological risk (AUC: 0.857).

**Graphical Abstract:**

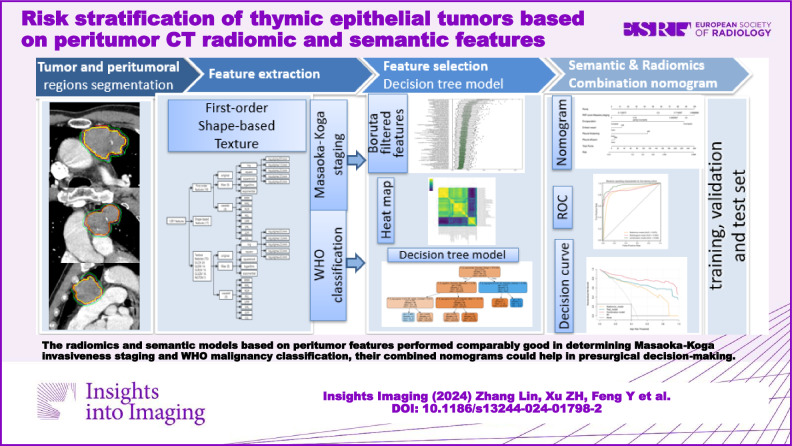

## Introduction

Thymic epithelial tumors (TET), a common primary tumor of the anterior mediastinum, are potentially malignant due to their propensity for local invasiveness and distant metastasis [1]. Histopathological classification of TET can be used to inform postoperative decisions such as neoadjuvant treatment strategies. The most important prognostic factors for TET are tumor stage and invasiveness [2–6]. Therefore, a non-invasive assessment of tumor staging and histological type prior to surgery and/or treatment is essential for optimal therapeutic outcomes.

The Masaoka–Koga staging and the World Health Organization (WHO) classification are commonly used systems for risk stratification of TET based on invasiveness and histopathology, respectively [7]. Semantic features associated with these two systems, i.e., clinical and radiological findings, are commonly used to determine the surgical approach to TET [8], particularly the choice of subxiphoid video thoracoscopic or conventional surgery [9]. Radiomics mines high-dimensional quantitative medical image data to reflect the underlying tumor characteristics and aid in decision-making [10]. Radiomics studies on CT images of TET have shown that imaging features within the delineated tumor region can assess malignant risk [11–16], and determine clinical staging [17–19]. However, due to the invasive nature of TET, the edges are often ill-defined, making accurate segmentation of tumor images difficult. Studies have shown that for other invasive-prone cancers, such as gastric cancer [20], pancreatic cancer [21], and meningioma [22, 23], the integration of intratumor and peritumor radiomics features outperformed only intratumor features in predicting tumor invasiveness. Furthermore, models that incorporated semantic features into both the intratumor and peritumor regions can create nomograms, that were more accurate in determining the invasiveness of meningioma [22].

It is hypothesized that the peritumor region of TET contains extensive underlying information related to tumor invasion. Therefore, this study aimed to develop a comprehensive nomogram integrating radiomics and semantic features selected from the intratumor and peritumor regions of TET to estimate the risk stratification for Masaoka–Koga staging and WHO classification.

## Methods

The local Institutional Review Board approved this retrospective study and waived patient consent.

### Study sample

**Fig. 1:**
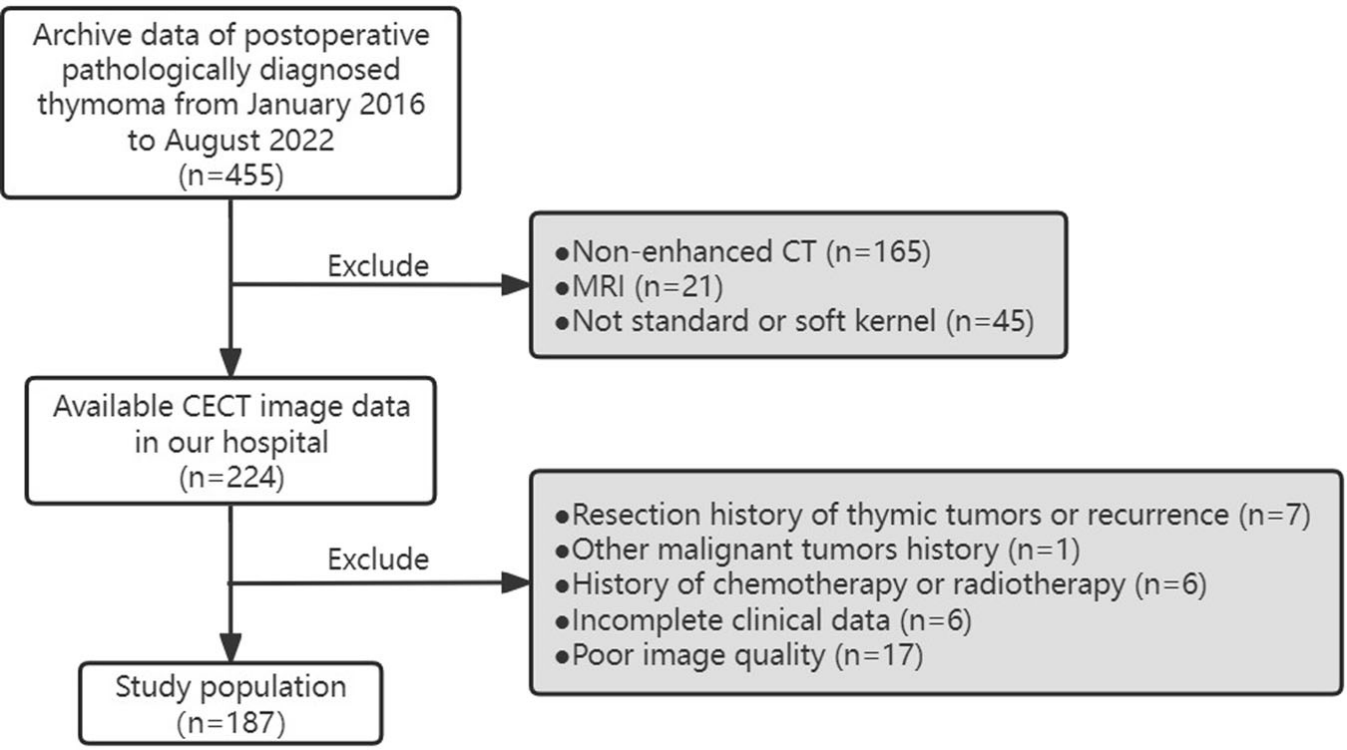
Patient inclusion flowchart

This retrospective study included consecutive cases of TET diagnosed between January 2016 and August 2022 at three hospitals: A-Shanghai General Hospital, B-Shanghai Songjiang Hospital, and C-Shanghai Jiangqiao Hospital. Histopathological findings in all cases were derived from surgical resection of the tumor, not by percutaneous biopsy. The inclusion criteria were as follows: (1) patients underwent thin-slice (< 1 mm) contrast-enhanced CT with standard or soft reconstruction kernel; (2) underwent tumor resection within two weeks after CT scanning and were diagnosed as TET by hematoxylin-eosin and immunohistochemistry staining; and (3) with complete clinical information and surgical records. Exclusion criteria were: (1) history of thymic tumor resection or tumor recurrence; (2) other malignancies; (3) chemotherapy or radiotherapy; and (4) poor-quality CT images due to artifacts. Figure 1 illustrates the inclusion flowchart.

### Grouping

The medical records of each patient were reviewed by two experienced oncologists to obtain a concordant judgment for Masaoka–Koga staging and WHO classification. A dichotomous classification was used based on clinical significance, i.e., early-stage (stages I and II) and advanced-stage (III and IV) invasiveness according to Masaoka–Koga staging, and low-risk (types A, AB, and B1) and high-risk (B2, B3, and thymic carcinoma) according to WHO classification. The training set consisted of two-thirds of the cases at Hospital A, with the remaining one-third assigned to the internal validation set. The external test set consisted of cases at Hospitals B and C.

### Semantic assessment

Two radiologists with 15 years and 10 years of experience in chest imaging, blinded to clinical information, assessed the semantic features of each case. A senior radiologist with 30 years of experience assessed interobserver agreement. The semantic features included two demographic, 13 tumor descriptive, 14 peritumor descriptive, and two symptomatic features (Table 1).

**Table 1:** Semantic features of TET

Demographic information	Tumor description features	Peritumor description features
• Age • Gender • Symptoms: none/chest tightness and discomfort/chest pain/cough or dyspnea/fever/edema/shoulder and back pain/others • Myasthenia gravis: none/yes	• Location: left/middle/right of the mediastinum • The maximum diameter of the tumor • Short diameter perpendicular to the maximum length diameter • Shape: oval/lobulated/irregular • Margin: smooth/rough • CT value after enhancement • Intratumor homogeneity after enhanced: homogeneous/slight heterogeneity/obvious heterogeneity • Enhancement degree (compared with chest wall muscle): higher/equal/lower • Cyst or necrosis: none/yes • Low attenuation area in tumor: less than 50%/more than 50% • Calcification: none/concentration/multifocal • Internal septa: none/yes • Encapsulation (adjacent mediastinal fat layer blurred or disappeared, or the presence of small nodules or cusp-like processes at the tumor edge): complete/partial incomplete/incomplete	• Fat space with pericardium or vessel: exists/disappear • Fat space with lung: exists/disappear • Obvious invasion of adjacent tissues: none/yes • Embed vessel: none/yes • Vessel invasion (irregular wall or intraluminal soft tissue): none/yes • Pericardium invasion: none/pericardial thickening/pericardial effusion • Pleural invasion (adjacent pleural thickening or the irregular and rough interface with the lung): none/yes • Pleural effusion: none/yes • Lung invasion (irregular interface with cusp-like changes with an absent space between tumor and lung): none/yes • Enlargement of hilar or mediastinal lymph nodes (short diameter >10 mm): none/yes • Chest wall invasion: none/yes • Isolated pleural/pericardial nodules: none/yes • Lung metastasis: none/yes • Elevated hemidiaphragm: none/yes

### Image acquisition

Six CT systems (Revolution, HD750, and VCT, GE Healthcare, Milwaukee, USA; Somatom Force and Flash, Siemens, Erlangen, Germany; uCT, United Imaging, Shanghai, China) were used for scanning (Appendix Table 1). Following the administration of contrast medium (Omnipague 300 mg/mL, GE Healthcare) via the antecubital vein at a dose of 1.5 mL/kg and a flow rate of 3.0 mL/s, CT scanning was conducted to obtain submillimeter-thin images from the apex of the lung to below the diaphragm.

### Image segmentation

Two experienced radiologists with > 10 years of experience (Readers 1 and 2) segmented tumors and quantified the segmented volume of interest (VOI) using research software (Radiomics v1.2.6, Frontier, Syngo Via, Siemens Healthineers) [24]. Reader 1 segmented the VOI of all cases, while Reader 2 segmented 30 randomly selected cases. Interobserver agreement was calculated based on these 30 cases.

A semi-automated approach was used to segment the tumor VOI (Appendix Fig. 1). First, the tumor VOI was segmented using an automated tool and then manually adjusted on a slice-by-slice basis to clearly outline the tumor edges. Second, peeling VOIs extending 3 mm, 5 mm, and 8 mm outwards were automatically segmented based on the tumor region identified in the first step. The volume between the outer boundary and the tumor edge was considered the peritumor region. The tumor and peritumor regions were inspected and corrected, and the bone component was manually removed from the VOIs. The segmentation process took 30 min per case.

### Radiomics modeling

**Fig. 2:**
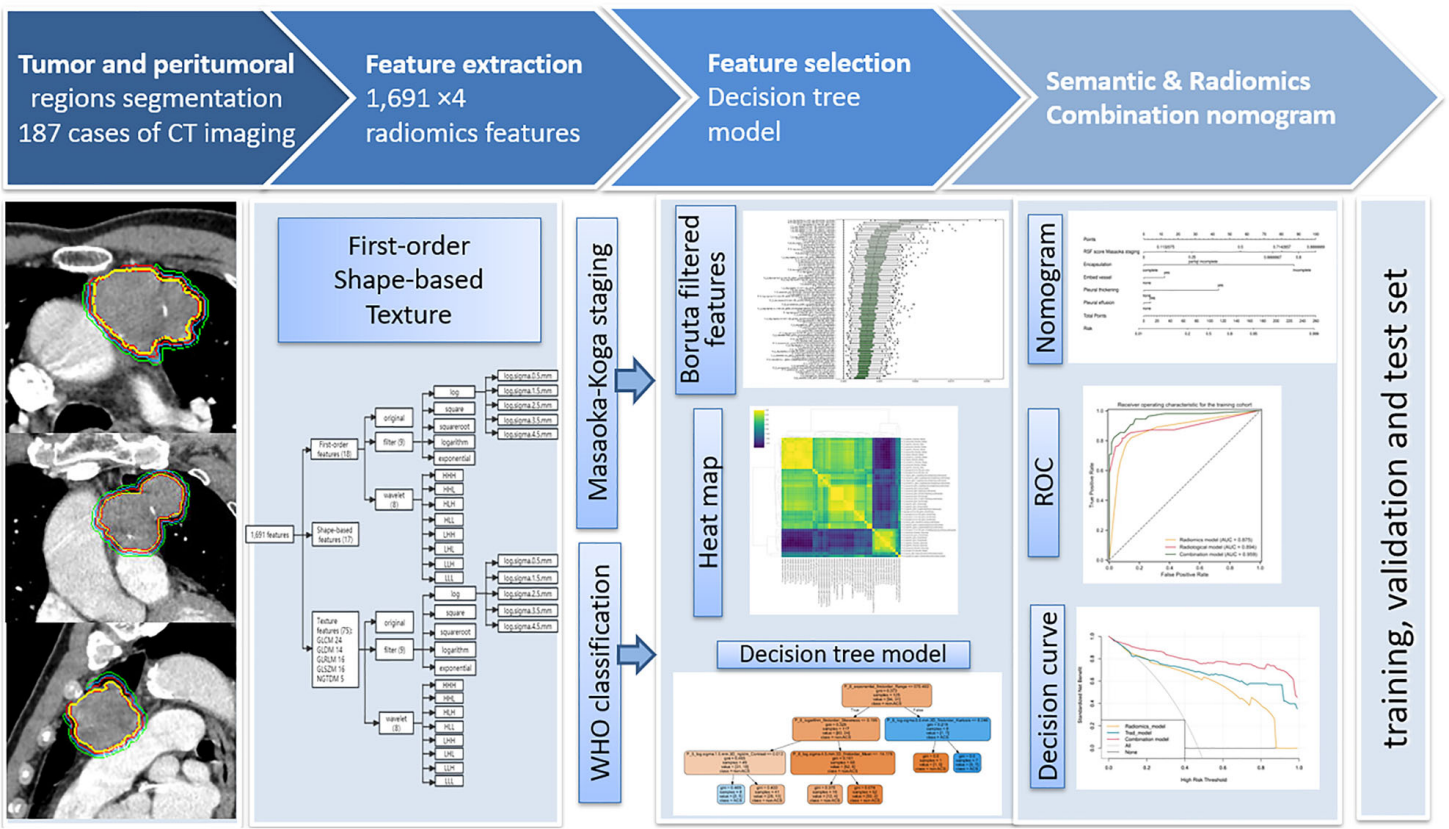
Four steps to build radiomics models: tumor and peritumor segmentation, feature extraction, feature selection, and model building

The construction of the radiomics model consisted of four steps: tumor and peritumor segmentation, feature extraction, feature selection, and model construction (Fig. 2). Feature extraction was conducted using the Pyradiomics library (v3.0, https://pyradiomics.readthedocs.io/en/latest/) and followed the Image Biomarker Standardization Initiative [25]. Appendix Fig. 2 illustrates the feature composition and feature classification. Four VOIs were extracted for each lesion, namely the tumor, and tumor extension (peritumor) at distances of 3 mm, 5 mm, and 8 mm. This resulted in a total of 1691 × 4 radiomics features per tumor.

Feature selection and model construction were performed for two classification systems, Masaoka–Koga staging and WHO classification. Radiomics features were selected in four steps. First, interobserver interclass coefficients (ICC) were calculated based on 30 randomly selected cases, and unstable and irreducible features with an ICC < 0.8 were excluded. Second, features that were significantly correlated (*p* < 0.05) with the true labels were screened by *F*-test. Third, the Boruta algorithm [26] was employed for 100 iterations to identify the most important features. Finally, Spearman’s correlation coefficient analysis with hierarchical clustering was applied to remove redundant features.

Three models were developed for each of the Masaoka–Koga staging and WHO classifications, including a radiomics feature model (named the radiomics model), a semantic feature model (semantic model), and a combined model that integrated radiomics scores and semantic features.

The radiomics model was constructed using a decision tree algorithm with 5-fold cross-validation parameter tuning via grid search method on the training set. This algorithm is highly interpretable and intuitively supports decision-making [27, 28]. It has been extensively used to classify large amounts of data in clinical studies [29–31]. The importance of features in the radiomics model was assessed by Gini impurity using the decision tree. The radiomics model generated a score between 0 and 1, indicating a trend towards bi-directional classification. A higher score indicates a higher likelihood of an advanced tumor stage.

The semantic model employed univariate logistic regression to select semantic features associated with Masaoka–Koga staging and WHO classification. Only features with a *p*-value < 0.05 in univariate analyses were included in the multivariate logistic regression model. Stepwise regression with the minimum Akaike information criterion was used as the model determination criterion.

The combined model was constructed using multivariate logistic regression, integrating the radiomics scores and the semantic features selected by the semantic models. All models were trained and built from the training set. For better visualization, nomograms of the combined model were generated. Each factor in the nomogram was assigned a quantitative score, and the total score was summed to calculate the risk of being highly invasive or malignant. A higher total score indicates a higher risk for the patient.

### Statistics

The numerical variables were described using the mean and standard deviation. Normally and non-normally distributed continuous variables were compared using the independent samples *t*-test and the Mann–Whitney *U*-test, respectively. The discriminative performance of the model was evaluated using the receiver operating characteristic (ROC) curve, area under the curve (AUC), accuracy, sensitivity, specificity, positive predictive value (PPV), and negative predictive value (NPV). The AUC of different models was compared using the Delong test [32]. The optimal cut-off for each model was determined by Youden’s index in the training set and applied to the test set. Calibration curves with Brier score and decision curve analysis were implemented to demonstrate the goodness of fit and clinical efficacy of different models.

A two-tailed *p* < 0.05 was considered statistically significant. The statistical analysis was conducted using software packages (SPSS v22.0, IBM; R v4.0.2, www.rstudio.com; Python v3.7, www.python.org) (Appendix Table 2).

## Results

### Study sample

Of 224 candidate patients, 187 were included (56.5 years ± 12.3, 101 men). In the training and validation set (*n* = 125), Masaoka–Koga staging yielded 70 patients with early stage and 55 with advanced stage; WHO classification yielded 94 with low risk and 31 with high risk. In the external test set (*n* = 62), Masaoka–Koga staging yielded 29 with early stage and 33 with advanced stage, and WHO classification yielded 47 with low risk and 15 with high risk. There were no significant differences (*p* > 0.05) between the training/validation and test sets in terms of demographic and semantic characteristics (Appendix Table 3).

### Radiomics features selection and significance analysis

One thousand, six hundred ninety-one radiomics features were extracted from each of the four regions, namely the tumor, 3-mm, 5-mm, and 8-mm extended peritumor regions. After ICC analysis, 1263, 940, 1095, and 1253 stable features were selected for each region (Appendix Table 4).

**Fig. 3:**
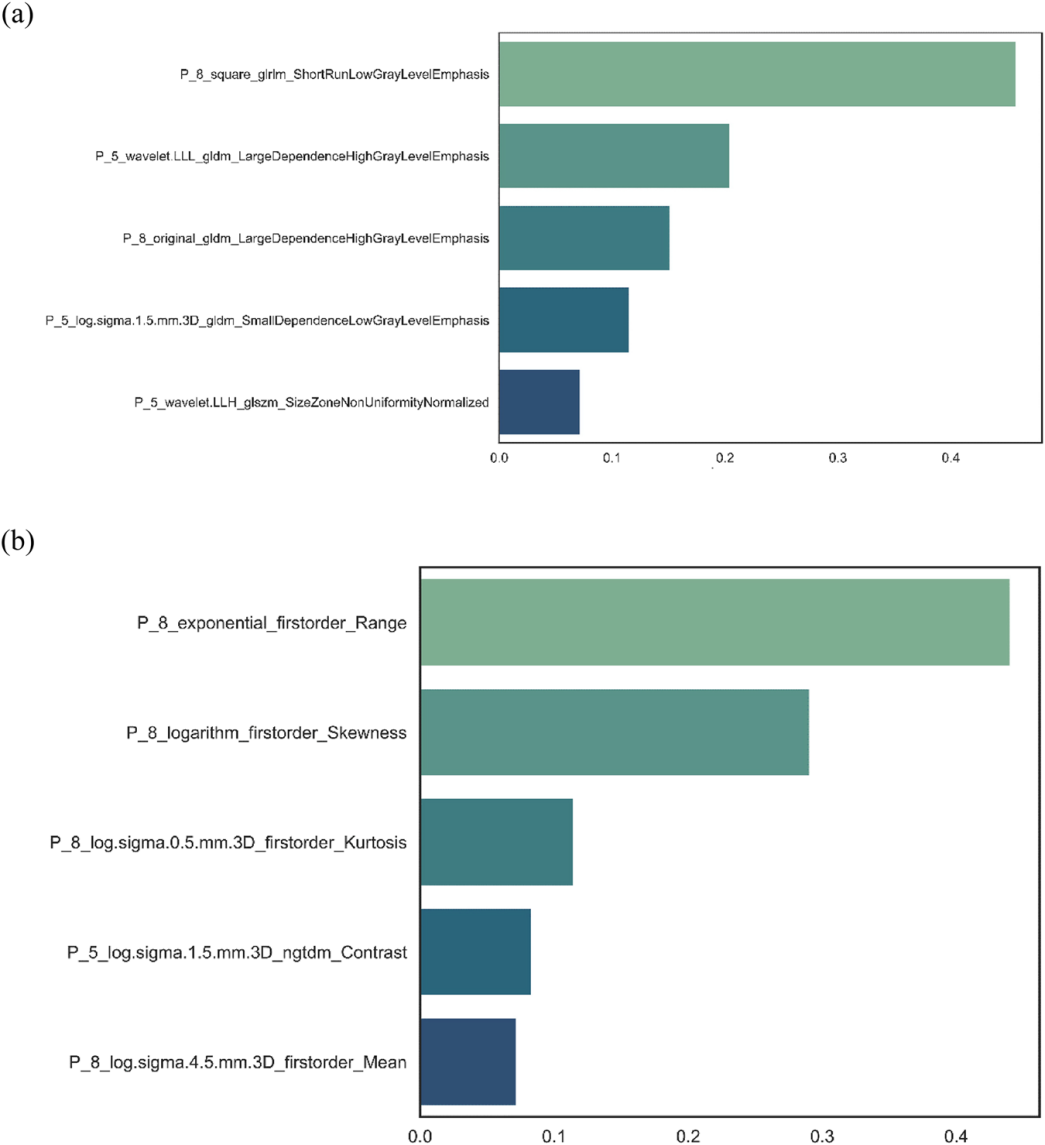
Five radiomics features included in the decision tree for Masaoka–Koga staging and WHO classification, the *y*-axis represented feature names and the *x*-axis represented feature importance, (**a**) five radiomics features for Masaoka–Koga staging and (**b**) five radiomics features for WHO classification

In the context of Masaoka–Koga staging, 336, 216, 352, and 378 features (1282 in total) were selected based on the *F*-test in the tumor, 3-mm, 5-mm, and 8-mm extended peritumor regions, respectively. From these features, the Boruta algorithm selected 1, 10, 19, and 16 features (46 in total), respectively (Appendix Fig. 3a). The Pearson correlation heatmap of the original features and selected features (Appendix Fig. 4a) demonstrates a low correlation between the selected 46 features. Following the removal of redundant features from the 46 features using the correlation matrix and clustering, the decision tree model contained only five features (three 5-mm and two 8-mm peritumors) (Fig. 3a and Appendix Fig. 5a).

For the WHO classification, 104, 257, 277, and 335 features (972 in total) were selected based on the *F*-test in the four regions, respectively. Subsequently, 1, 25, 19, and 33 features (78 in total) were selected (Appendix Fig. 3b). The Pearson correlation heatmap (Appendix Fig. 4b) displays the original features and the 78 selected features. Following the removal of redundant features, the decision tree model included five features (one 5-mm and four 8-mm peritumor) (Fig. 3b and Appendix Fig. 5b).

### Semantic feature selection and significance analysis

Using univariate logistic regression, we marked with asterisks the semantic features selected in the Masaoka–Koga staging and WHO classification (Appendix Table 5). Then, the features with a *p*-value < 0.05 in univariate logistic regression (11 features for Masaoka–Koga staging and 9 for WHO classification) were included in multivariate logistic regression. Semantic models were constructed using minAIC as the criterion. For Masaoka–Koga staging, four features (encapsulation partial loss [odd ratio (OR)1 = 1.38]/complete loss [OR2 = 14.50], embedding vessel [OR = 6.10], pleural thickening [OR = 11.67] and pleural effusion [OR = 9.72]) were selected to build the semantic model. Two features (margin [OR = 6.32] and adjacent invasion [OR = 5.39]) were selected for the WHO classification.

### Performance of radiomics, semantic, and combined models

**Fig. 4:**
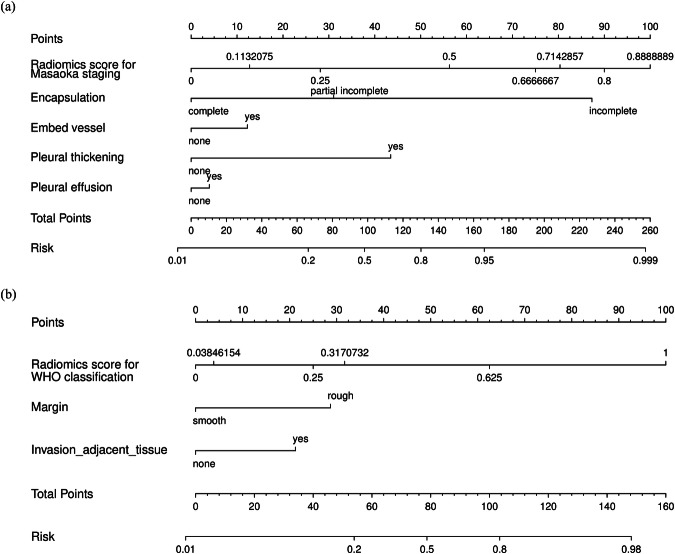
Nomogram combining semantic and radiomics features for (**a**) Masaoka–Koga staging and (**b**) WHO classification

The nomograms quantified the inputs and outputs of the combined model (Fig. 4). The calibration curves (Appendix Fig. 6) show that the predictive curves of the combined models are closer to the reference line (slope = 1) than those of the radiomics and semantic models, indicating greater predictive ability.

**Fig. 5:**
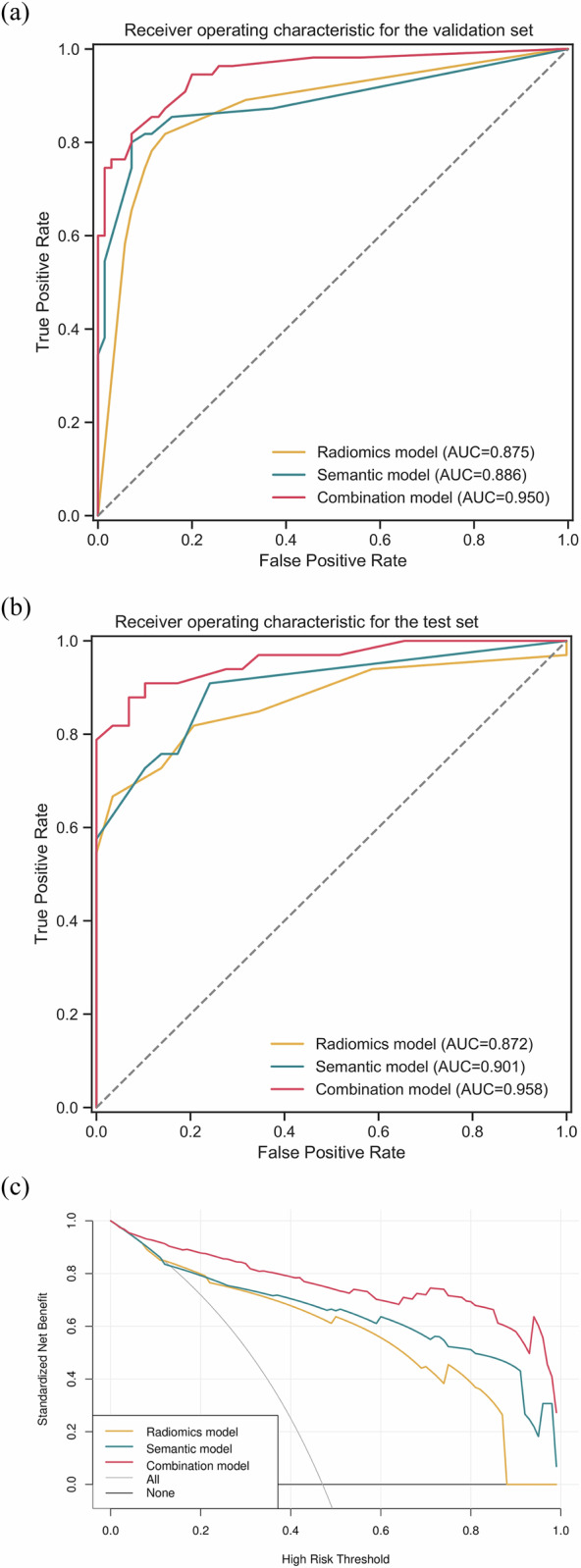
Performance of the semantic, radiomics, and combined models for Masaoka–Koga staging, (**a**) ROC curve of the validation set, (**b**) and (**c**) decision ROC curve of the test set, and (**c**) decision curve analysis (DCA) curve of the validation and test sets

In the test set, the combined model for Masaoka–Koga staging achieved the highest AUC of 0.958 (95% confidence interval [CI]: 0.912–1.000), significantly higher than 0.872 (0.781–0.963) of the radiomics model (DeLong’s *p* = 0.045) and 0.901 (0.828–0.974) of the semantic models (*p* = 0.047) (Table 2 and Fig. 5a, b). The AUC of the radiomics model was comparable to that of the semantic model (*p* = 0.627). The combined model demonstrated accuracy, sensitivity, specificity, PPV, and NPV of 0.871, 0.818, 0.931, 0.931, and 0.818, respectively, in which accuracy, specificity, PPV, and NPV were significantly higher than those of the radiomics and semantic models (*p* < 0.05).

**Table 2:** Performance comparison of the semantic, radiomics, and combined models for Masaoka–Koga staging

	Radiomics model		Semantic model		Combined model	
	Validation	Test	Validation	Test	Validation	Test
AUC	0.875	0.872	0.886	0.901	0.950	0.958^a,b^
95% CI (DeLong)	(0.811–0.938)	(0.781–0.963)	(0.823–0.950)	(0.828–0.974)	(0.912–0.987)	(0.912–1.000)
Youden index	0.375		0.610		0.520	
Accuracy	0.840	0.758	0.872	0.807^a^	0.880	0.871^a,b^
95% CI	(0.764–0.899)	(0.633–0.858)	(0.801–0.925)	(0.686–0.896)	(0.810–0.931)	(0.762–0.943)
Sensitivity	0.818	0.849	0.800	0.727	0.818	0.818
Specificity	0.857	0.655	0.929	0.897^a^	0.929	0.931^a,b^
PPV	0.818	0.737	0.898	0.889^a^	0.900	0.931^a,b^
NPV	0.857	0.792	0.855	0.743	0.867	0.818^a,b^
Comparisons of validation and test (DeLong’s test) *p*-value	0.961		0.772		0.788	

*AUC* area under curve, *95% CI* 95% confidence interval, *PPV* positive predictive value, *NPV* negative predictive value

^a^ Significant difference with the radiomics model

^b^ Significant difference with semantic model

**Fig. 6:**
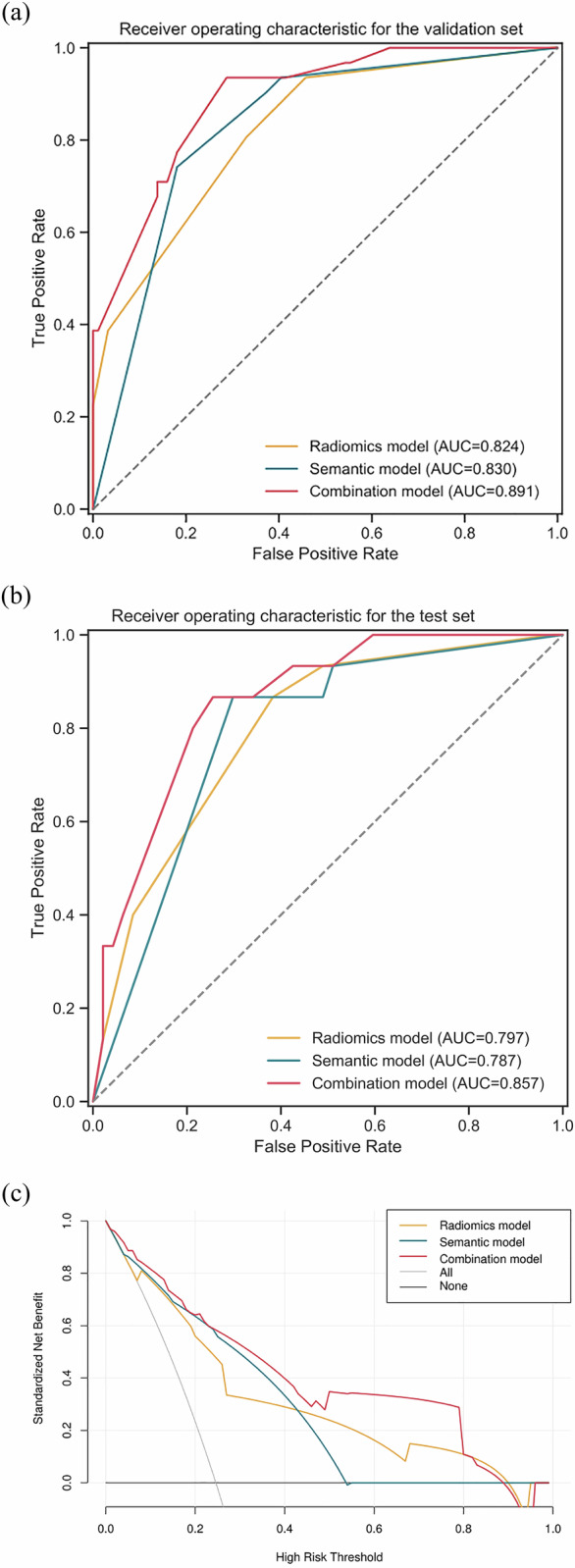
Performance of the semantic, radiomics, and combined models for WHO classification, (**a**) ROC curve of the validation set, (**b**) ROC curve of the test set, and (**c**) decision curve analysis (DCA) curve of the validation and test sets

In the test set, the combined WHO classification model also showed the highest AUC of 0.857 (0.760–0.955) (Table 3 and Fig. 6a, b), higher than 0.797 (0.680–0.914) of the radiomics model (*p* = 0.143) and 0.787 (0.676–0.899) of the semantic models (*p* = 0.152). The AUC of the radiomics model was comparable to that of the semantic model (*p* = 0.903).

**Table 3:** Performance comparison of the semantic, radiomics, and combined models for WHO classification

	Radiomics model		Semantic model		Combined model	
	Validation	Test	Validation	Test	Validation	Test
AUC	0.824	0.797	0.831	0.787	0.891^a,b^	0.857
(95% CI)	(0.746–0.903)	(0.680–0.914)	(0.757–0.904)	(0.676–0.899)	(0.830–0.951)	(0.760–0.955)
Youden index	0.144		0.394		0.179	
Accuracy	0.640	0.613	0.800	0.742	0.768	0.645
(95% CI)	(0.549–0.724)	(0.481–0.734)	(0.719–0.866)	(0.615–0.845)	(0.684–0.839)	(0.513–0.763)
Sensitivity	0.936	0.933	0.742	0.867	0.936	0.933
Specificity	0.543	0.511	0.819	0.702	0.713	0.553
PPV	0.403	0.378	0.575	0.482	0.518	0.400
NPV	0.962	0.960	0.906	0.943	0.971	0.963
Comparisons of validation and test (DeLong’s test) *p*-value	0.709		0.527		0.574	

*AUC* area under curve, *95% CI* 95% confidence interval, *PPV* positive predictive value, *NPV* negative predictive value

^a^ Significant difference with the radiomics model

^b^ Significant difference with semantic model

The decision analysis curves demonstrate that the combined model provides a greater net clinical benefit than the radiomics and semantic models in Masaoka–Koga staging (Fig. 5c). When selecting a predictive probability between 25% and 80%, the combined model offers a higher net clinical benefit than the other models in the WHO classification (Fig. 6c).

## Discussion

In this study, a machine learning-based decision tree model was used to effectively classify peritumor radiomics features and semantic features of TET. The radiomics model, comprising the features most contributing to Masaoka–Koga staging and WHO classification, showed that peritumor features were predictive of advanced invasiveness and high malignancy risk of TET. The radiomics model and the semantic model demonstrated comparable predictive performance, while the combined nomograms exhibited higher performance (AUC = 0.96 for Masaoka–Koga staging and 0.86 for WHO classification).

Contrast-enhanced CT is the most used imaging method for preoperative evaluation of TET. Unlike needle biopsy, non-invasive CT carries no risk of complications or tumor seeding. Furthermore, a presurgical needle biopsy does not necessarily represent the entire tumor [33]. CT results are helpful in making presurgical decisions and personalizing treatment plans. Complete surgical resection of high-risk or advanced TET is less likely than for low-risk or early-stage TET, necessitating preoperative preparation to determine indications for minimally invasive surgery [34] or multimodal therapy [35, 36]. The clinical significance of classifying TET is greater than that of thymoma alone, as it is difficult to differentiate between thymoma and thymic carcinoma preoperatively, and clinical decisions are made without histological results. However, histological heterogeneity between thymoma and thymic carcinoma may affect texture analysis when analyzed together [12, 19].

TET is highly heterogeneous with varying radiological appearances, histopathological features, and prognosis. Depending on the histological type, the five subgroups of thymoma resemble spindle cell tumor, lymphoma or carcinoma. Low-risk thymoma (types A, AB, and B1) exhibits thymus-like structures, whereas epithelial-dominated thymoma (high-risk) lose these structures to varying degrees, and thymic carcinomas exhibit features of squamous cell carcinoma, adenocarcinoma or undifferentiated carcinoma [37]. For these reasons, radiomics features within the tumor, such as intensity, texture, and wavelets, may have a limited contribution to risk or invasion classification. This study involved a four-step feature selection (ICCs, *F*-test, Boruta algorithm, and Spearman’s correlation) where the number of selected intratumor features was gradually reduced until they were eliminated. Eventually, five peritumoral features were selected as the most important features and were incorporated into two decision tree models, which also confirms that the use of peritumor features predicts Masaoka–Koga staging and WHO classification better than only intratumor features [11, 14, 15]. These features provide important information about the tumor margin. Jeong et al [38] reported that the tumor contour, mediastinal fat, and large vessel invasion are useful in distinguishing WHO classification subgroups. Other subjective CT-based studies have shown that a number of features containing shape [39] and surrounding infiltration [40], as well as intratumor features, are helpful for WHO classification [8, 41]. We believe that peritumor radiomics has the advantage of extracting higher-order boundary features of TET with rough and fuzzy edges, and is better than intratumor analysis in WHO stratification.

Masaoka–Koga staging is important for presurgical decision-making, and the peritumor region contains valuable information that is highly relevant to tumor invasiveness. For other lesions such as lung nodules, combining intranodular radiomics with the perinodular region improved the performance in differentiating lung adenocarcinoma from granuloma over intranodular radiomics alone [42]. Incorporating brain-tumor interface radiomics with intratumor radiomics performed well in predicting brain invasion of meningioma [23]. Shape, capsule integrity, and vascularity grading were semantic features independently associated with transcapsular invasion of TET [43]. We involved intratumor and peritumor features and finally selected five peritumor features to construct a well-performing radiomics model (AUC = 0.87 for Masaoka–Koga staging and 0.80 for WHO classification). Our results suggest that tumor boundary and adjacent information play a crucial role in Masaoka–Koga staging.

Although radiomics models were effective, they lacked the information provided by semantic models, such as the association with adjacent tissue beyond 8 mm or bone, distant lung parenchyma, and pleural changes. Our semantic model included four features in the Masaoka–Koga staging model and two features in the WHO classification model. This suggests that TET with rough margins, adjacent invasion, and pleural changes were indicative of advanced stage or high risk, rather than intratumor features (size, calcification, internal density, and low attenuation area). This finding is consistent with the principle of Masaoka–Koga staging based on tumor invasiveness. In the context of WHO classification, previous studies have also demonstrated the utility of peritumor features in risk prediction. For instance, lesion margins and infiltration were significantly associated with high-risk TET [35]. Lobulated or irregular contours, mediastinal fat invasion, and vascular invasion were more prevalent in high-risk TET [34]. As for reproducibility, Park et al [43] suggested that semantic CT features such as shape, capsule integrity, and vascularity grading of TET are reproducible. In our study, the selected peritumor semantic features demonstrated a significant contribution to the stratification of TET, and the nomograms combining radiomics and semantic features exhibited optimal performance.

There are some limitations. First, due to the limited sample size, we performed two-way classification. With a larger sample size, multilayer classification would be possible. Second, the sample size was unbalanced between risk strata, and more advanced or high-risk cases are needed to strengthen the model. Third, differences in CT equipment, scanning parameters, and image reconstruction algorithms would reduce confidence, but would increase the generalizability of the model. Although this retrospective study used standardized scans, such as similar slice thicknesses, slice spacing, and reconstruction kernels, where possible, an ideal standardized approach may improve model performance. Fourth, a prospective study is needed to validate the effectiveness of our proposed model for clinical decision-making.

## Conclusion

The integration of peritumor radiomics features and semantic features into nomograms has the potential to improve the accuracy of invasiveness grading and risk stratification of TET.

## Supplementary information

ELECTRONIC SUPPLEMENTARY MATERIAL

## Data Availability

The datasets used and/or analyzed during the current study are available from the corresponding author upon reasonable request.

## Abbreviations

AUC: Area under the curve
CI: Confidence interval
ICC: Interobserver interclass coefficient
NPV: Negative predictive value
OR: Odd ratio
PPV: Positive predictive value
ROC: Receiver operating characteristic
TET: Thymic epithelial tumors
WHO: World Health Organization
