# Supplementary material for: Risk stratification of thymic epithelial tumors based on peritumor CT radiomics and semantic features
Source: Insights Imaging. 2024 Oct 22;15:253. doi: 10.1186/s13244-024-01798-2 (PMC11496418; doi:10.1186/s13244-024-01798-2)
Supplement: Supplementary file 1 — ELECTRONIC SUPPLEMENTARY MATERIAL [file 13244_2024_1798_MOESM1_ESM.pdf]

Risk stratification of thymic epithelial tumors based on peritumor CT radiomics and semantic features

ELECTRONIC SUPPLEMENTARY MATERIAL

Appendix figures:

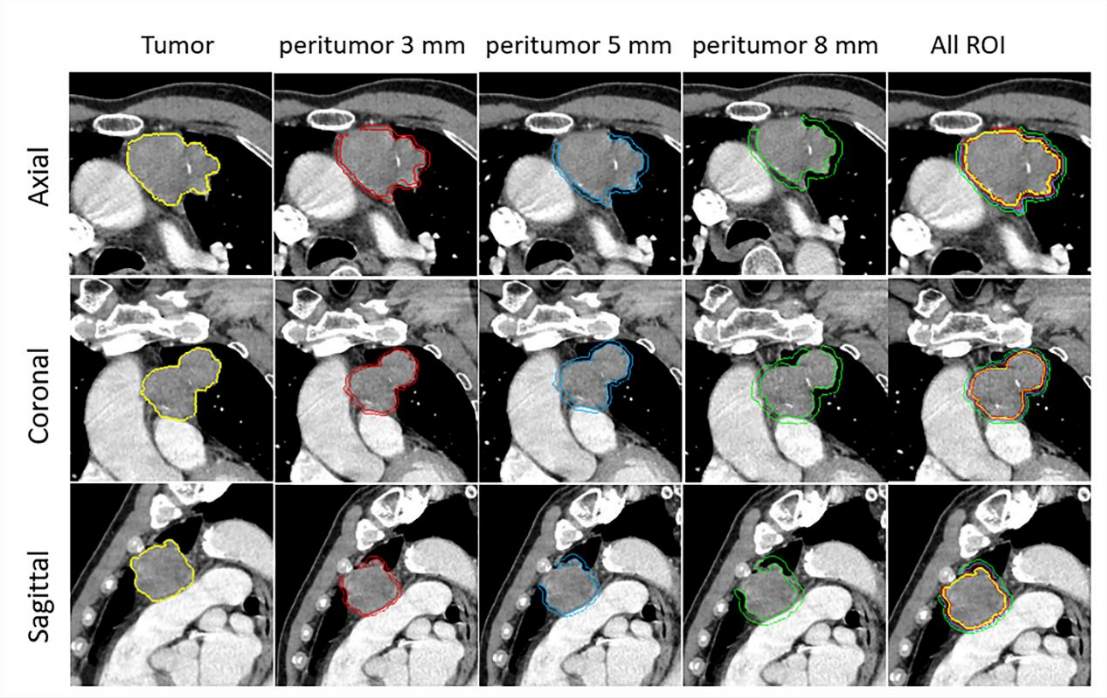

Appendix Figure 1. Volume of interest (VOI) of the segmented and delineated tumor and the VOI of the peritumor regions expanding 3 mm, 5 mm, and 8 mm from the tumor margin. In order to evaluate the relationship between the tumor and the surrounding soft tissues, the VOI includes adjacent fat, heart, blood vessels, and lymph nodes, etc. Bones were manually removed to avoid interference.

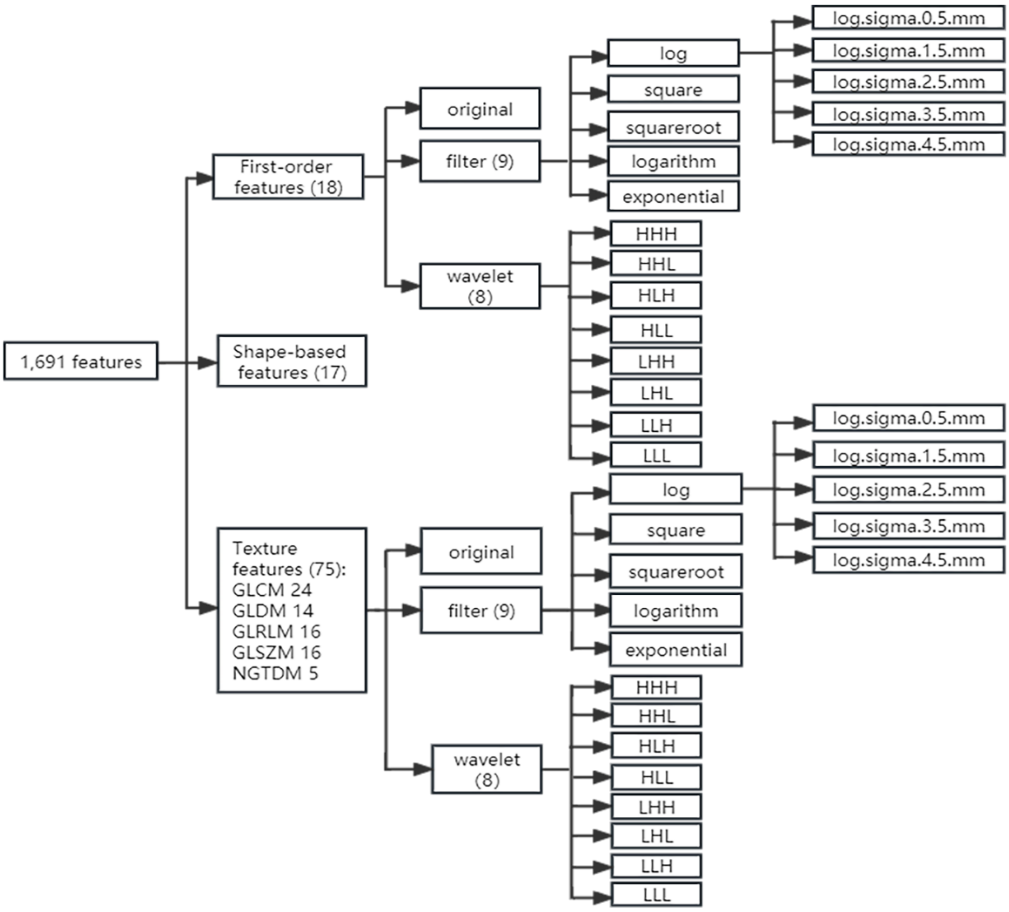

Appendix Figure 2. The composition and classification of radiomics features. Three types of radiomics features are automatically extracted, including 17 shape-based, 18 first-order, and 75 texture features. The 75 texture features include the following groups: 24 gray-level co-occurrence matrix (GLCM), 14 gray-level dependence matrix (GLDM), 16 gray-level run-length matrix (GLRLM), 16 gray-level size zone matrix (GLSZM), and 5 neighboring gray-tone difference matrix (NGTDM) features. To further mine the data, wavelet transforms and nonlinear transforms are used for texture features and first-order histogram features. The wavelet transform can be used to mine image

information by decomposing the images at different frequencies in 3 directions (x, y, z): HHH, HHL, HLH, HLL, LHH, LHL, LLH, LLL (H stands for high-frequency and L for low-frequency). The nonlinear transforms on image voxels include square, square root, logarithmic, and exponential operations. A total of 1691 features are extracted from each VOI. The extracted computational features are classified into original, filtered (9 types), and wavelet transformed (8 types) features.

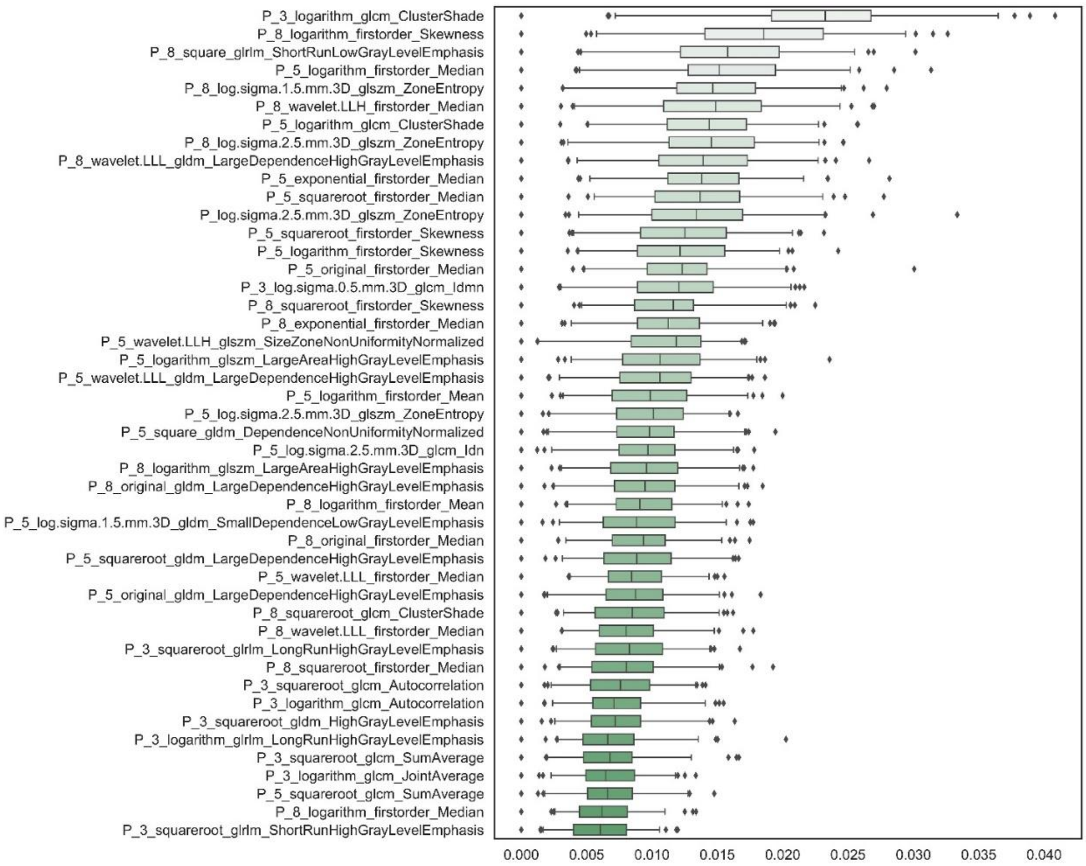

(a)

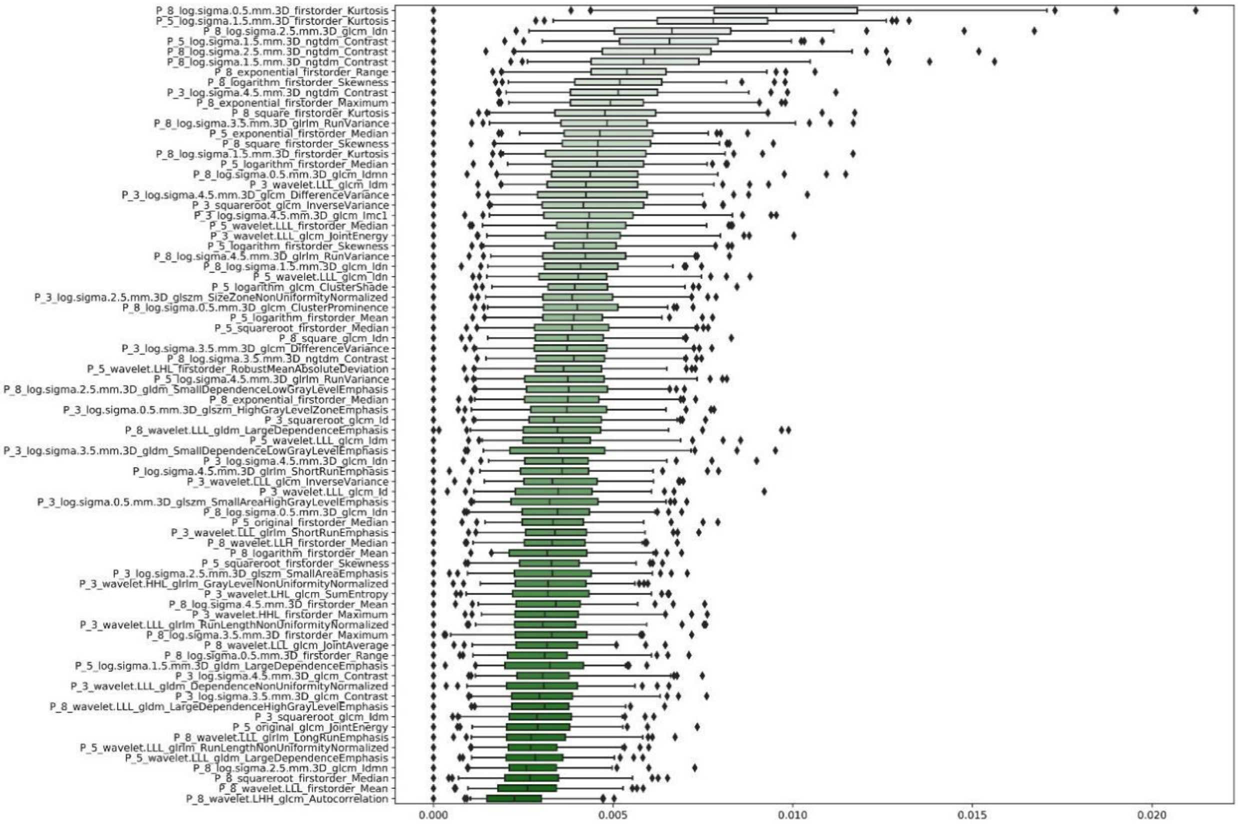

(b)

Appendix Figure 3. Boruta algorithm-filtered radiomics features and their corresponding importance, (a) 46 features selected for Masaoka-Koga staging, (b) 78 features for World Health Organization classification.

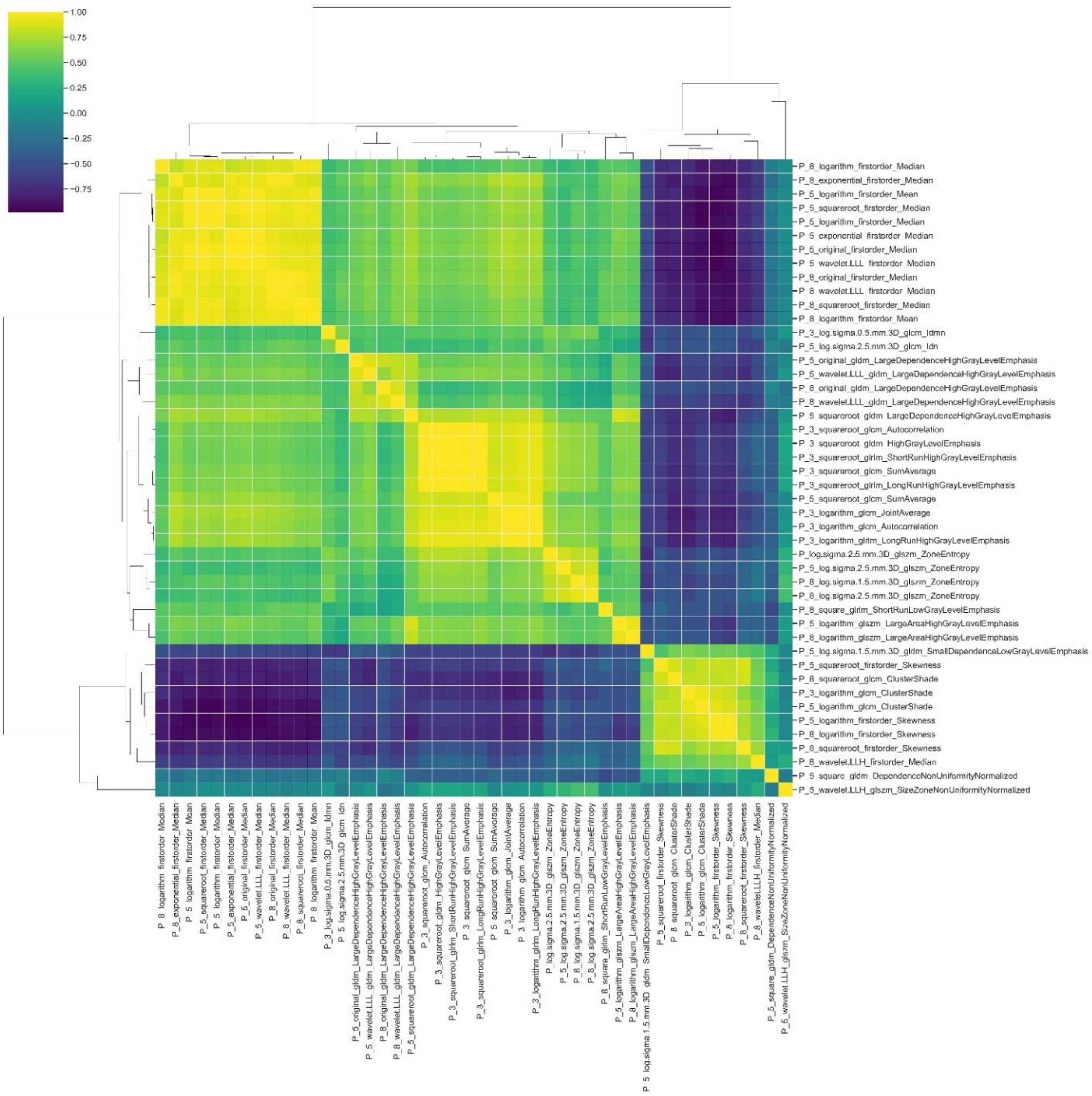
1.00
0.75
0.50
0.25
0.00
−0.25
−0.50
−0.75
P_8_logarithm_firstorder_Median
P_8_exponential_firstorder_Median
P_5_logarithm_firstorder_Mean
P_5_squareroot_firstorder_Median
P_5_logarithm_firstorder_Median
P_5_exponential_firstorder_Median
P_5_original_firstorder_Median
P_5_wavelet.LLL_firstorder_Median
P_8_original_firstorder_Median
P_8_wavelet.LLL_firstorder_Median
P_8_squareroot_firstorder_Median
P_8_logarithm_firstorder_Mean
P_3_log.sigma.0.5.mm.3D_glcm_Idmn
P_5_log.sigma.2.5.mm.3D_glcm_Idn
P_5_original_gldm_LargeDependenceHighGrayLevelEmphasis
P_5_wavelet.LLL_gldm_LargeDependenceHighGrayLevelEmphasis
P_8_original_gldm_LargeDependenceHighGrayLevelEmphasis
P_8_wavelet.LLL_gldm_LargeDependenceHighGrayLevelEmphasis
P_5_squareroot_gldm_LargeDependenceHighGrayLevelEmphasis
P_3_squareroot_glcm_Autocorrelation
P_3_squareroot_gldm_HighGrayLevelEmphasis
P_3_squareroot_glrlm_ShortRunHighGrayLevelEmphasis
P_3_squareroot_glcm_SumAverage
P_3_squareroot_glrlm_LongRunHighGrayLevelEmphasis
P_5_squareroot_glcm_SumAverage
P_3_logarithm_glcm_JointAverage
P_3_logarithm_glcm_Autocorrelation
P_3_logarithm_glrlm_LongRunHighGrayLevelEmphasis
P_log.sigma.2.5.mm.3D_glszm_ZoneEntropy
P_5_log.sigma.2.5.mm.3D_glszm_ZoneEntropy
P_8_log.sigma.1.5.mm.3D_glszm_ZoneEntropy
P_8_log.sigma.2.5.mm.3D_glszm_ZoneEntropy
P_8_square_glrlm_ShortRunLowGrayLevelEmphasis
P_5_logarithm_glszm_LargeAreaHighGrayLevelEmphasis
P_8_logarithm_glszm_LargeAreaHighGrayLevelEmphasis
P_5_log.sigma.1.5.mm.3D_gldm_SmallDependenceLowGrayLevelEmphasis
P_5_squareroot_firstorder_Skewness
P_8_squareroot_glcm_ClusterShade
P_3_logarithm_glcm_ClusterShade
P_5_logarithm_glcm_ClusterShade
P_5_logarithm_firstorder_Skewness
P_8_logarithm_firstorder_Skewness
P_8_squareroot_firstorder_Skewness
P_8_wavelet.LLH_firstorder_Median
P_5_square_gldm_DependenceNonUniformityNormalized
P_5_wavelet.LLH_glszm_SizeZoneNonUniformityNormalized

(a)

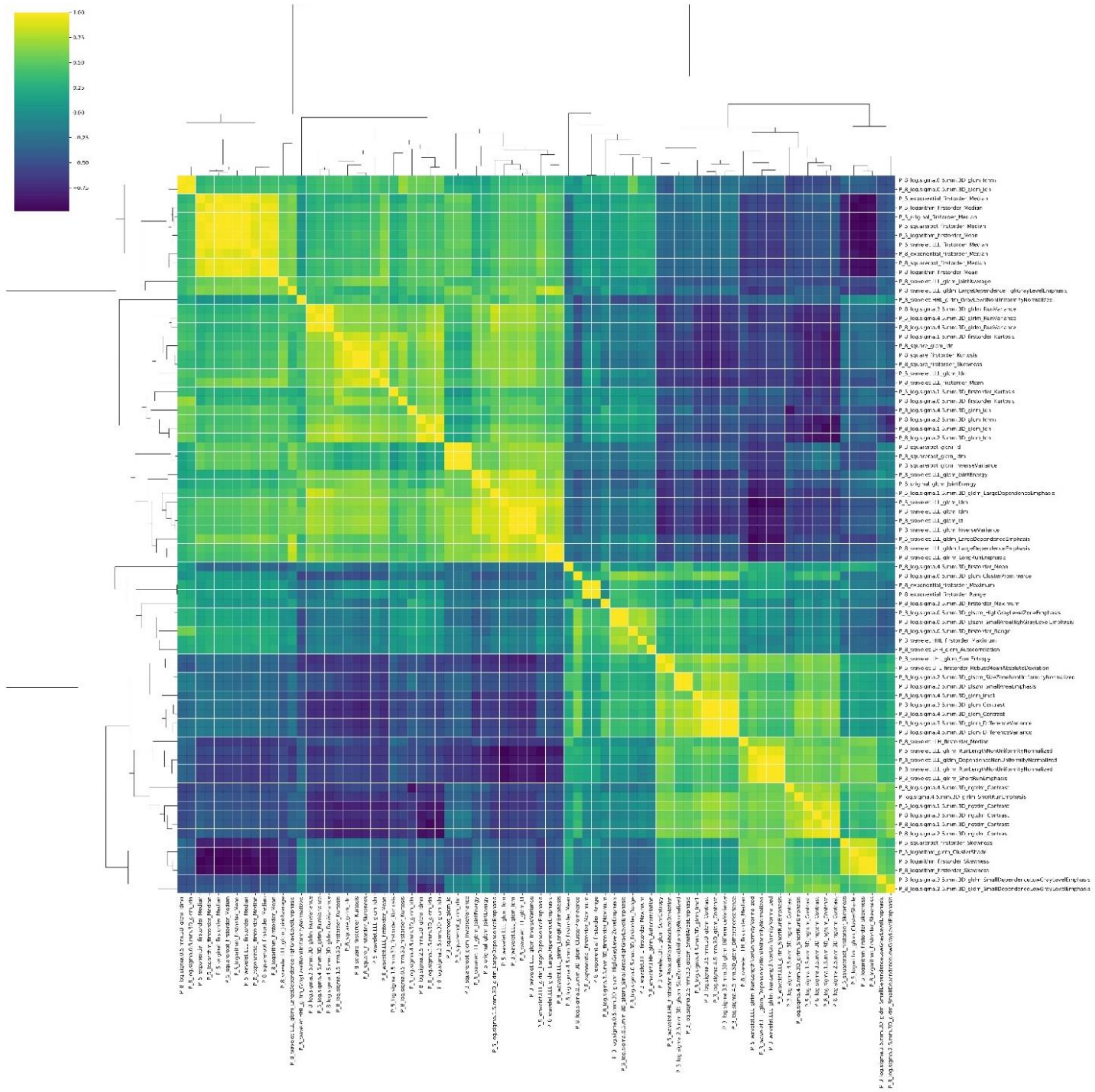

(b)

Appendix Figure 4. Pearson correlation heat maps of the original features and the selected features, (a) 46 tumor and peritumor features for Masaoka-Koga staging, (b) 78 tumor and peritumor features for World Health Organization classification.

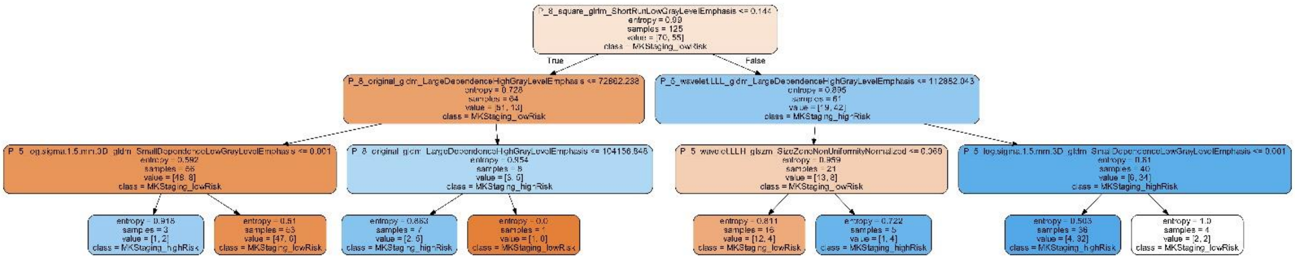

(a)

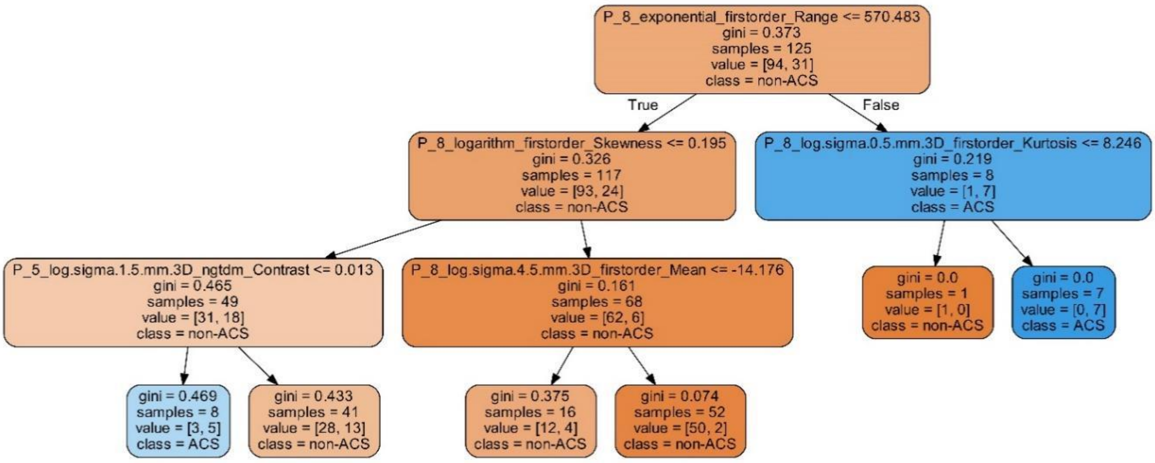

(b)

Appendix Figure 5. Decision tree models for (a) Masaoka-Koga staging and (b) World Health Organization classification.

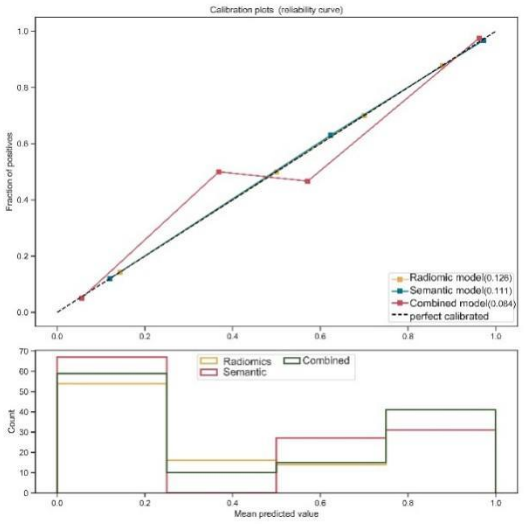

(a)

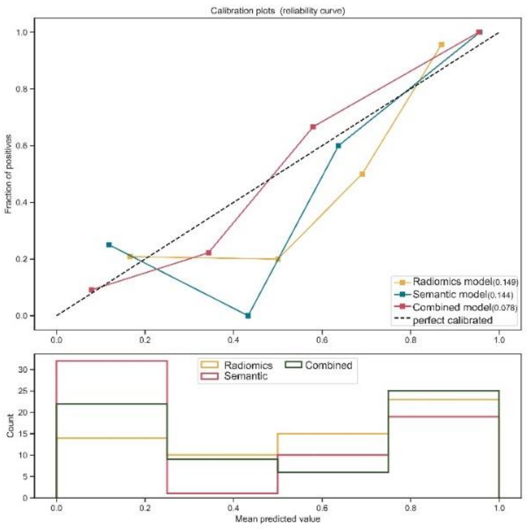

(b)

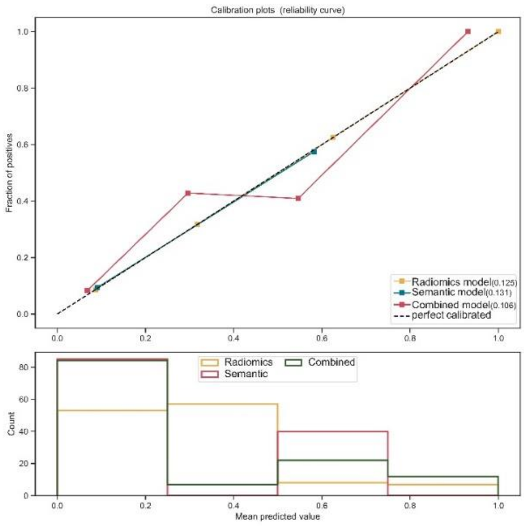

(c)

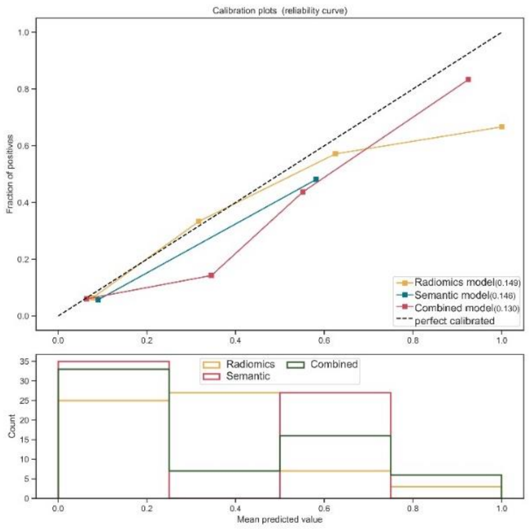

(d)

Appendix Figure 6. Calibration curves of (a) Masaoka-Koga staging of the validation set, (b) Masaoka-Koga staging of the test set, (c) World Health Organization classification of the validation set and (d) World Health Organization classification of the test set.

Appendix tables:

Appendix Table 1. Image acquisition and reconstruction protocol of CT scanners

	GE Revolution	GE HD750	Siemens Force	Siemens Flash	GE VCT	UCT
**Acquisition mode**	Helical	Helical	Helical	Helical	Helical	Helical
**Tube voltage, kVp**	120	120	100	100	120	120
**Tube current, mAs**	50-200 (smart mode)	50-200 (smart mode)	75 (quality reference)	100 (effective)	50-110 (smart mode)	150-200
**Dose modulation**	on	on	on	on	on	on
**Collimation, mm**	256×0.625	64×0.625	192×0.6	128×0.6	64×0.625	64×0.6
**Pitch**	0.984	0.984	0.95	0.95	0.984	0.9
**Rotation time, ms**	500	500	500	500	500	500
**Reconstruction kernel**	Standard, lung	Standard, lung	B70f,B30	B70f,B30	Standard, lung	B_soft B_sharp
**Field of view, mm**	350	350	350	350	350	350
**Slice thickness, mm**	0.625	0.625	0.75	0.75	0.625	0.5
**Slice increment, mm**	0.625	0.625	0.75	0.75	0.625	0.5

Appendix Table 2. Software packages used in this study

Statistical analysis	Packages	Version	Web
Reliability test: intraclass correlation coefficient	‘irr in R	0.84.1	https://cran.r-project.org/web/packages/irr/index.html
Feature selection: Boruta	‘Boruta’ in Python	0.3	https://pypi.org/project/Boruta/
Feature selection: Correlation	‘sicpy’ in Python	1.6.0	https://www.scipy.org/
Model establishment: Decision Tree and Grid search with Cross validation	‘scikit-learn’ in Python	0.13.2	https://scikit-learn.org/stable/
Model performance evaluation: Calibration curve and Brier score calculation	‘scikit-learn’ in Python	0.13.2	https://scikit-learn.org/stable/
model establishment: multivariate logistic regression	‘rms’ in R	6.0-1	https://CRAN.R-project.org/package=rms
Model performance evaluation: decision curve analysis	‘rmda’ in R	1.6	https://CRAN.R-project.org/package=rmda
Model performance evaluation: Delong Test and 95%CI for ROC curve analysis	‘pROC’ in R	1.16.2	https://CRAN.R-project.org/package=pROC

Appendix Table 3. Semantic information of thymic epithelial tumors in the training and validation set and the test set of Masaoka-Koga staging and WHO classification

Semantic features	Description	Training and validation set	Test set	*p*. overall
		N=125	N=62	
Gender	female	55 (44.0%)	31 (50.0%)	0.536
	male	70 (56.0%)	31 (50.0%)	
Age		56.0 [46.0;62.0]	57.5 [51.0;63.0]	0.102
Location	left	27 (21.6%)	13 (21.0%)	0.995
	middle	54 (43.2%)	27 (43.6%)	
	right	44 (35.2%)	22 (35.5%)	
Diameter long		44.8 [30.3;66.5]	43.4 [31.7;65.5]	0.889
Diameter short		28.8 [19.8;41.1]	31.5 [20.8;43.6]	0.442
Shape	oval	29 (23.2%)	22 (35.5%)	0.202
	lobulated	46 (36.8%)	20 (32.3%)	
	irregular	50 (40.0%)	20 (32.3%)	
Margin	smooth	62 (49.6%)	26 (41.9%)	0.405
	rough	63 (50.4%)	36 (58.1%)	
CT value after enhancement		73.0 [63.0;91.0]	73.5 [62.0;86.5]	0.709
Intratumor homogeneity after enhanced	homogeneous	52 (41.6%)	31 (50.0%)	0.361
	slight heterogeneous	45 (36.0%)	16 (25.8%)	
	obvious heterogeneous	28 (22.4%)	15 (24.2%)	
Enhancement degree (compared with muscle)	higher	64 (51.2%)	28 (45.2%)	0.502
	equal	54 (43.2%)	32 (51.6%)	
	lower	7 (5.6%)	2 (3.2%)	
Cyst or necrosis	none	88 (70.4%)	39 (62.9%)	0.386
	yes	37 (29.6%)	23 (37.1%)	

Low attention area	less than 50%/	112 (89.6%)	57 (91.9%)	0.805
	more than 50%	13 (10.4%)	5 (8.1%)	
Calcification	none	98 (78.4%)	43 (69.4%)	0.085
	concentration	6 (4.8%)	9 (14.5%)	
	multifocal	21 (16.8%)	10 (16.1%)	
Internal septa	none	119 (95.2%)	60 (96.8%)	1.000
	yes	6 (4.8%)	2 (3.2%)	
Encapsulation	complete	55 (44.0%)	27 (43.6%)	0.753
	partial incomplete	47 (37.6%)	26 (41.9%)	
	incomplete	23 (18.4%)	9 (14.5%)	
Fat space with pericardium or vessel	exists	84 (67.2%)	35 (56.5 %)	0.240
	disappear	41 (32.8%)	27 (43.6%)	
Fat space with lung	exists	19 (15.2%)	13 (21.0%)	0.610
	disappear	106 (84.8%)	49 (79.0%)	
Obvious adjacent invasion	none	81 (64.8%)	33 (53.2%)	0.171
	yes	44 (35.2%)	29 (46.8%)	
Embed vessel	none	96 (76.8%)	43 (69.4%)	0.358
	yes	29 (23.2%)	19 (30.6%)	
Vessel invasion	none	105 (84.0%)	47 (75.8%)	0.175
	yes	20 (16.0%)	15 (24.2%)	
Pericardium invasion	none	88 (70.4%)	42 (67.7%)	0.929
	pericardial thickening	19 (15.2%)	10 (16.1%)	
	pericardial effusion	18 (14.4%)	10 (16.1%)	
Lung invasion	none	94 (75.2%)	45 (72.6%)	0.797
	yes	31 (24.8%)	17 (27.4%)	
Pleural thickening	none	77 (61.6%)	36 (58.1%)	0.759
	yes	48 (38.4%)	26 (41.9%)	
Pleural effusion	none	113 (90.4%)	58 (93.6%)	0.655
	yes	12 (9.6%)	4 (6.5%)	
Chest wall invasion	none	120 (96.0%)	57 (91.9%)	0.304
	yes	5 (4.0%)	5 (8.1%)	
Lymph nodes enlargement	none	111 (88.8%)	57 (91.9%)	0.681
	yes	14 (11.2%)	5 (8.1%)	
Isolated pleural/ pericardial nodules	none	119 (95.2%)	58 (93.6%)	0.733
	yes	6 (4.8%)	4 (6.5%)	
Lung metastasis	none	121 (96.8%)	60 (96.8%)	1.000
	yes	4 (3.2%)	2 (3.2%)	
Myasthenia gravis	none	117 (93.6%)	56 (90.3%)	0.556
	yes	8 (6.4%)	6 (9.7%)	
Elevated hemidiaphragm	none	124 (99.2%)	62 (100. 0%)	1.000
	yes	1 (0.8%)	0 (0.0%)	
Symptoms	none	92 (73.6%)	43 (69.4%)	0.578

	yes	33 (26.4%)	19 (30.6%)	

p.overall represented the statistical difference of the semantic information between the training and validation set and the test set.

Appendix Table 4. Interclass coefficients in the volume of interests of tumor, peritumor regions extended 3 mm, 5 mm, and 8 mm

Interclass coefficient	**Tumor**	**Peritumor 3 mm**	**Peritumor 5 mm**	**Peritumor 8 mm**
0-0.5	158	176	90	55
0.5-0.8	266	573	505	380
0.8-1.0	1263	940	1095	1253

Appendix Table 5. Univariate logistic regression for semantic features

Semantic features	Masaoka-Koga staging			WHO classification		
	OR	p value	95%CI	OR	p value	95%CI
Diameter Long	1.0159	0.0192*	1.003-1.030	1.0042	0.5105	0.991-1.017
Diameter Short	0.9991	0.6678	0.992-1.003	0.9991	0.7393	0.987-1.003
Shape	2.3226	0.1488	0.777-7.958	2.4074	0.2139	0.660-11.524
Margin	8.9583	1.81E-07*	4.040-21.140	15.7333	1.87E-05*	5.102-69.217
Enhanced CT value	0.9822	0.0318*	0.966-0.998	0.9862	0.1489	0.967-1.004
Enhanced uniformity	1.4120	0.4011	0.632-3.181	1.3542	0.5147	0.543-3.408
Enhanced degree	1.4486	0.3217	0.697-3.031	0.9333	0.8756	0.388-2.211
Necrosis	0.8182	0.6136	0.371-1.774	0.6222	0.3261	0.227-1.544
Low attention area	0.7750	0.6715	0.222-2.470	0.5204	0.4133	0.078-2.088
Calcification	2.9000	0.2316	0.539-21.642	1.3148	0.7598	0.175-7.148
Internal septa	0.6226	0.5926	0.084-3.317	0.5933	0.6399	0.030-3.875
Encapsulation	6.6761	8.02E-05*	2.694-18.069	4.3714	0.0169*	1.395-16.658
Fat layer with vessel	6.260E-08	0.9909	NA-3.89E+121	2.38E-08	0.9904	NA-1.40E+121
Fat layer with Lung	7.480E-17	0.9913	0.000-5.48E+43	0.1111	0.0649	0.008-1.046
Invasion adjacent tissue	18.5000	2.86E-09*	7.438-51.972	12.6857	3.38E-07*	5.001-35.943
Embed vessel	20.0230	3.88E-06*	6.416-88.747	6.6462	5.07E-05*	2.694-17.012
Invasion vessel	36.4167	0.0006*	7.113-667.519	6.7895	0.0002*	2.480-19.613
Invasion pericardium	9.4500	0.0002*	3.092-	4.7571	0.0042*	1.628-14.039

			35.720			
Pleural thickening	26.3571	8.89E-11*	10.355-76.105	7.9350	1.15E-05*	3.259-21.108
Interface irregular	311362134.3	0.9871	0.000-3.30E+160	10.4476	1.33E-06*	4.135-28.022
Pleural effusion	17.2499928	0.0073*	3.190-320.833	2.3901	0.1645	0.660-8.129
Invasion chest wall	21911905.11	0.9874	0.000-NA	2.0920	0.4311	0.266-13.219
Lymph node enlargement	9.488372093	0.0043*	2.436-62.902	7.2818	0.0011*	2.287-25.791
Pleural/pericardium nodules	22359086.84	0.9862	0.000-NA	1.5517	0.6223	0.207-8.383
Lung Metastasis	21482259.91	0.9888	0.000-NA	9.9643	0.0503	1.222-205.824
Myasthenia gravis	0.4025	0.2771	0.057-1.830	6.52E-08	0.9906	NA-1.49E+44
Elevated hemidiaphragm	2745788.859	0.9866	0.000-NA	18041146.96	0.9908	0.000-NA
